# Gastrointestinal Symptoms in Morbid Obesity

**DOI:** 10.3389/fmed.2014.00049

**Published:** 2014-12-04

**Authors:** Mustafa Huseini, G. Craig Wood, Jamie Seiler, George Argyropoulos, Brian A. Irving, Glenn S. Gerhard, Peter Benotti, Christopher Still, David D. K. Rolston

**Affiliations:** ^1^Institute of Obesity, Geisinger Health System, Danville, PA, USA; ^2^Department of Biochemistry, Molecular Biology, Pathology and Laboratory Medicine, Pennsylvania State University, Hershey, PA, USA; ^3^Department of Internal Medicine, Geisinger Health System, Danville, PA, USA

**Keywords:** obesity, gastrointestinal, symptoms, body mass index, clinic-based studies

## Abstract

**Background:** Several reports have shown an increased prevalence of gastrointestinal (GI) symptoms in obese subjects in community-based studies. To better understand the role of the GI tract in obesity, and because there are limited clinic-based studies, we documented the prevalence of upper and lower GI symptoms in morbidly obese individuals in a clinic setting.

**Objective:** The aim of our study was to compare the prevalence of GI symptoms in morbidly obese individuals in a weight management clinic with non-obese individuals with similar comorbidities as morbidly obese individuals in an Internal Medicine clinic.

**Methods:** Class II and III obese patients BMI >35 kg/m^2^ (*N* = 114) and 182 non-obese patients (BMI <25 kg/m^2^) completed the GI symptoms survey between August 2011 and April 2012 were included in this study. The survey included 24 items pertaining to upper and lower GI symptoms. The participants rated the frequency of symptoms as absent (never, rarely) or present (occasionally, frequently). The symptoms were clustered into five categories: oral symptoms, dysphagia, gastroesophageal reflux, abdominal pain, and bowel habits. Responses to each symptom cluster were compared between obese group and normal weight groups using logistic regression.

**Results:** Of the 24 items, 18 had a higher frequency in the obese group (*p* < 0.005 for each). After adjusting for age and gender, the obese patients were more likely to have upper GI symptoms: any oral symptom (OR = 2.3, *p* = 0.0013), dysphagia (OR 2.9, *p* = 0.0006), and any gastroesophageal reflux (OR 3.8, *p* < 0.0001). Similarly, the obese patients were more likely to have lower GI symptoms: any abdominal pain (OR = 1.7, *p* = 0.042) and altered bowel habits (OR = 2.8, *p* < 0.0001).

**Conclusion:** These observations suggest a statistically significant increase in frequency of both upper and lower GI symptoms in morbidly obese patients when compared to non-obese subjects.

## Introduction

The obesity epidemic continues unabated world-wide. The increasing prevalence of overweight and obesity has resulted in 3.4 million deaths, 3.9% of years lost, and 3.8% of disability adjusted life-years world-wide. Greater than 50% of obese individuals reside in the USA, China, India, Russia, Brazil, Egypt, Germany, Pakistan, and Indonesia (1). The USA accounted for 13% of these obese individuals in 2013. Obesity trends in the USA along with associated comorbidities could, for the first time result in decreased life expectancy (2). The Centers for Disease Control, National Center for Health Statistics in the USA, has estimated that, between 2011 and 2012, more than one-third of adults (34.9%) were obese and that two-thirds of the US population is overweight or obese (3). The burden of obesity and its associated health problems have a significant adverse impact on the US healthcare system. In 2008, the annual healthcare cost in the US was estimated to be a staggering $ 147 billion (4). Besides cost, obesity results in 100,000 excess deaths per annum (5) and estimated years of life lost (difference between the number of years an individual would live if not obese and the number of years expected to live if obese) was estimated to be 13 years for white men aged 20 years with BMI >45 kg/m^2^ and 8 years for white women aged 20–30 years with BMI >45 kg/m^2^ compared to normal weight controls (6).

The gastrointestinal (GI) tract is normally the only site of nutrient and calorie absorption. Improved understanding of the role of the GI tract in obesity may help develop strategies to decrease obesity and obesity-related health costs. Because there are limited clinic-based studies, in contrast to population-based studies, we documented the prevalence of upper and lower GI symptoms in morbidly obese individuals attending the Obesity Institute outpatient clinic and normal weight individuals with similar comorbidities attending the General Internal Medicine (GIM) clinic at the Geisinger Medical Center in Danville, PA, USA.

## Subjects and Methods

This study included morbidly obese patients (BMI >35 kg/m^2^) with metabolic comorbidities or BMI >40 kg/m^2^ and normal weight patients (BMI 18.5–24.9 kg/m^2^) who sought care at Geisinger Medical Center, Danville, PA, USA. All potential study subjects were asked to complete the GI Symptoms survey, which was developed by the study team to address the frequency of GI symptoms. The questionnaire included 24 items and was formulated via literature review, review of gastroenterology textbooks and incorporated the Rome II and III questionnaires. The patients rated the frequency of symptoms as “Never, Rarely, Occasionally, and Frequently” (Table 1). The symptoms were categorized into the following five clusters: (1) oral symptoms – mouth ulcers, bleeding gums, dental problems, tongue swelling, sour taste; (2) dysphagia – gagging, trouble swallowing solids or liquids; (3) reflux – sour taste, nausea, vomiting, heartburn, belching/burping; (4) abdominal pain – cramping with hunger or post-meal; (5) bowel habits – diarrhea, fecal urgency and incontinence, bloating, constipation, excessive flatus, use of laxatives, rectal bleeding.

**Table 1:** **GI symptom survey responses by symptom category (114 obese patients and 182 normal weight patients)**.

	Group	Never (%)	Rarely (%)	Occasionally (%)	Frequently (%)	*p*-value
**ORAL SYMPTOMS**						
Do you get painful mouth ulcers?	Normal	57	34	9	1	0.355
	C II and III obese	59	36	5	0	
Do you get sore or swollen tongue?	Normal	88	10	2	1	0.015
	C II and III obese	76	18	4	1	
How often do you get dental work like filling treatments, root canal treatments, etc?	Normal	16	63	17	3	0.570
	C II and III obese	18	56	23	4	
Do you get bleeding gums?	Normal	51	36	12	2	0.0003
	C II and III obese	36	36	18	11	
Do you often get sensation of bitter or sour taste in your mouth?	Normal	77	15	6	2	<0.0001
	C II and III obese	58	19	15	8	
**DYSPHAGIA**						
Do you experience trouble swallowing liquid food?	Normal	87	7	3	2	0.538
	C II and III obese	79	17	4	0	
Do you have trouble swallowing solid foods?	Normal	75	15	7	2	0.047
	C II and III obese	66	18	14	3	
Do you experience gagging or frequent need to clear throat?	Normal	65	23	9	3	0.0010
	C II and III obese	50	24	16	11	
**REFLUX**						
How often do you experience nausea?	Normal	29	55	11	5	0.0093
	C II and III obese	21	46	27	6	
Do you vomit or throw up after eat*ing*?	Normal	88	10	2	0	0.218
	C II and III obese	83	13	4	0	
How often do you experience heartburn also called reflux?	Normal	36	41	14	10	<0.0001
	C II and III obese	22	30	28	20	
Do you often get sensation of bitter or sour taste in your mouth?	Normal	77	15	6	2	<0.0001
	Obese	58	19	15	8	
How often do you get belching or burping?	Normal	22	46	21	12	<0.0001
	C II and III obese	10	29	32	29	
**ABDOMINAL PAIN**						
Do you get abdominal pain, discomfort or cramping within 2 h of eating?	Normal	58	25	9	9	0.010
	C II and III obese	40	30	23	7	
Do you experience abdominal pain, discomfort or cramping when you are hungry?	Normal	67	21	9	3	0.0017
	C II and III obese	49	27	19	5	
**BOWEL HABITS**						
How often do you get diarrhea (more than three stools in a day) or stools with loose consistency?	Normal	27	53	13	6	<0.0001
	C II and III obese	16	38	29	18	
Do you get fecal urgency (having to rush to have a bowel movement?	Normal	41	44	10	5	<0.0001
	C II and III obese	21	33	31	15	
Have you ever lost control of your stools?	Normal	82	13	4	1	<0.0001
	C II and III obese	57	32	8	4	
Do you underclothes get soiled by feces?	Normal	81	14	5	0	0.0002
	C II and III obese	63	24	8	5	
Do you often get constipation (less than three bowel movements per week)?	Normal	45	31	13	11	0.771
	C II and III obese	45	33	13	9	
Do you get abdominal bloating?	Normal	35	38	14	13	0.0014
	C II and III obese	22	28	35	15	
How often have you had rectal bleeding in last 1 year?	Normal	74	18	6	2	0.0014
	C II and III obese	58	19	19	3	
Do you pass excessive flatus?	Normal	28	36	21	15	0.0005
	C II and III obese	15	28	31	26	
How would you rate your use of laxatives or enema?	Normal	82	13	4	1	0.724
	C II and III obese	85	10	2	3	

The obese study group included patients with extreme obesity that agreed to participate in a separate, ongoing research study of bariatric surgery within the Nutrition and Weight Management Clinic of the Obesity Institute (7). Consecutive patients that completed the GI Symptoms survey between August 2011 and April 2012 were included in this research study (*N* = 114). During the same time period, non-obese patients were recruited from the GIM clinic. For this study group, potential participants were identified by querying the Geisinger electronic health record for patient’s seen in the GIM clinic in the preceding 1 year with a BMI 18.5–25 kg/m^2^. The study population was limited to those aged 18–64 years in both groups. Patients with a prior history of intra-abdominal surgery, inflammatory bowel disease, any acute infectious processes, and patients using orlistat were excluded from the study. The eligible normal weight patients with comorbidities similar to the obese group (*N* = 652) were mailed a packet that included an introduction letter, the GI Symptoms survey, and a pre-addressed, pre-paid return envelope. Four patient letters were returned due to wrong address. Of the remaining 648 patients, 140 returned the survey within 6 weeks. The remaining patients were sent a second packet with a revised introduction letter and the same survey and envelope. This resulted in an additional 42 completed surveys, and an overall total of 182 normal weight participants (overall completion rate of 28%).

Responses to each symptom question were compared between the normal weight and obese group using Cochran–Armitage trend tests. For each of the five symptom domain groups, patients were classified as having any symptom within the group (defined as “Occasionally” or “Frequently” for any of the questions within the symptom domain group) or no symptoms (defined as “Never” or “Rarely” for all of the questions within the symptom group). In bivariate analysis, the percent of patients with any symptom was compared between the normal weight and obese groups using a chi-square test. In multivariate analysis (adjusting for age and gender), logistic regression was used to measure the association between the presence of any symptom and obesity status. Separate regression models were used for each symptom group. To confirm that categorizing the likert scaled response items into present (i.e., “Occasionally” or “Frequently”) or absent (i.e., “Never” or “Rarely”) did not influence the results, the data were reanalyzed using the likert scale for each question. The categorized version is presented because these simpler results agreed with the more complex analysis. SAS version 9.3 was used for statistical analysis and a *p*-value <0.05 was considered significant.

Funding for this study was provided through Geisinger internal funds. The Geisinger Institutional Research Review Board approved the study.

## Results

The 182 normal weight patients had a mean BMI of 22.1 kg/m^2^ (SD = 1.7), a mean age of 50.8 years (SD = 11.1), and 78% were female (Table 2). As compared to the 470 non-responders, the 182 normal weight patients that responded to the survey were older (50.8 years versus 45.0 years, *p* < 0.0001) and more likely to be female (78 versus 70% female, *p* = 0.040). The 114 class II obese patients had a mean BMI of 50.1 kg/m^2^ (SD = 10.0, range: 35.0 kg/m^2^–78.3 kg/m^2^), a mean age of 44 years (SD = 10.8), and 79% were female.

**Table 2:** **Description of the normal weight and obese study populations**.

		Normal weight	Class II and III obese
		*N* = 182	*N* = 114
Gender	Male	22% (*n* = 40)	21% (*n* = 24)
	Female	78% (*n* = 142)	79% (*n* = 90)
Age	Mean (SD)	50.8 (11.1)	44.0 (10.8)
	Range (min, max)	(20, 64)	(19, 64)
BMI	Mean (SD)	22.1 (1.7)	50.1 (10.0)
	Range (min, max)	(18.5, 25.0)	(35.0, 78.3)

Of the 24 individual symptom questions, 18 had a higher frequency in the class II and III obesity group as compared to the normal weight group (*p* < 0.05, Table 1). Significant associations were found for three of the five oral symptom questions, two of the three dysphagia questions, four of the five reflux questions, both of the abdominal pain questions, and seven of the nine bowel habits questions.

**Figure 1:**
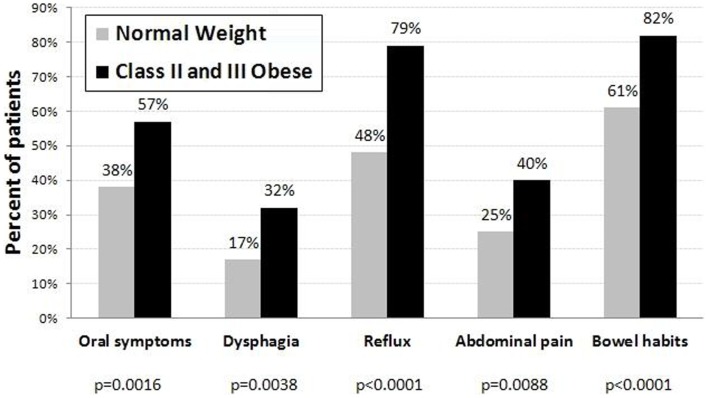
**Percent of normal weight (*N* = 182) and class II and III obese (*N* = 114) patients with occasional/frequent symptoms in each symptom group**.

When combining questions into symptom groups, the prevalence of any symptom within each of the five groups was higher in the class II obese group (Figure 1). Oral symptoms were present in 57% of the obese group versus 38% of the normal weight patients (*p* = 0.0016). Dysphagia symptoms were present in 32% of the obese group versus 17% in those with normal weight (*p* = 0.0038). Reflux was significantly higher in obese patients compared to normal weight patients (79 versus 48%, *p* < 0.0001). Forty percent of the obese subjects experienced abdominal pain symptoms as compared to 25% of the normal weight patients (*p* = 0.0088). Finally, altered bowel habits were more common in the obese group (82%) as compared to the normal weight group (61%, *p* < 0.0001).

After adjusting for age and gender, the prevalence of symptoms remained higher in the class II obese group for all of the symptom groups (Table 3). Those in the obese group were 2.28 times more likely to have oral symptoms [95% CI = (1.38, 3.78), *p* = 0.0013], 2.90 times more likely to have dysphagia [95% CI = (1.58, 5.33), *p* = 0.0006], 3.77 times more likely to have reflux [95% CI = (2.16, 6.59), *p* < 0.0001], 1.73 times more likely to have abdominal pain [95% CI = (1.02, 2.94), *p* = 0.042], and 2.79 times more likely to have altered bowel habits [95% CI = (1.55, 5.01), *p* < 0.0001].

**Table 3:** **Multivariate regression results for presence of any symptom within the symptom group**.

Symptom group	Adjusted^a^ OR for class II and III obesity	95% CI	*p*-value
Oral symptoms	2.28	1.38, 3.78	0.0013
Dysphagia	2.90	1.58, 5.33	0.0006
Reflux	3.77	2.16, 6.59	<0.0001
Abdominal pain	1.73	1.02, 2.94	0.042
Bowel habits	2.79	1.55, 5.01	0.0006

*^a^Adjusted for age and gender*.

## Discussion

Both population-based (8–10) and hospital- or office-based (11, 12) studies have demonstrated an increase in the prevalence of GI symptoms in obese individuals. The most extensively studied are esophageal symptoms, which include gastroesophageal reflux (often used interchangeably with heartburn), which also occurs in approximately 40% of individuals in the general population (13). Other esophageal symptoms include the regurgitation of solids or sour liquids, globus, chest pain, dysphagia, dry cough, and throat pain (8). Our study, which comprised morbidly obese individuals (mean BMI >50 kg/m^2^), confirms these findings. This is not surprising since individuals with a BMI >30 kg/m^2^ have a threefold increase in the odds of having reflux symptoms (14). This finding has been confirmed repeatedly including in the recent Progression of Gastroesophageal Reflux Disease study, which demonstrated that there was increased severe heartburn, regurgitation, and esophagitis with higher BMI, with regurgitation being more frequent than heartburn (15) and in two meta-analyses (16, 17). There was a trend toward greater GERD symptoms in individuals with a BMI >30 kg/m^2^ (pooled adjusted odds ratio of 1.94 compared to1.43 for individuals with a BMI of 25–30 kg/m^2^) when GERD was diagnosed by a validated questionnaire or endoscopy findings (16). Not only is heartburn more common in the obese individual (32.6 versus 18.8% in normal weight controls) so is endoscopic evidence of hiatal hernia and gastritis (11). In contrast, in massive obesity no association with heartburn was found (18).

The mechanism of upper GERD symptoms in obesity remains conjectural partly because there are limited studies addressing this (13). While basal lower esophageal sphincter (LES) pressure is similar in morbidly obese individuals and normal weight individuals (19), the frequency of transient lower esophageal sphincter relaxation (TLESR) is increased with increasing BMI and waist circumference (20), suggesting that waist circumference plays a role in GERD in obesity. Additionally, increased intra-abdominal pressure (21), increased gastroesophageal pressure gradient, hiatal hernia (11), altered visceral sensitivity and increased gastric basal pressure (22) all probably play a variable role. We did not evaluate the effect of weight circumference and waist to hip ratio in our patients.

There are less clear mechanistic studies on GI symptoms such as chest pain, bloating, and post-prandial fullness. The role that pro-inflammatory cytokines play in obesity, i.e., TNF-α, IL-6, IL-1, and leptin (23) and the concomitant decrease in the pro-inflammatory cytokine, adiponectin (24) may play in esophageal contractility and increased incidence of esophagitis is unclear as is their role in gastric motility, gastritis, small and large bowel function in obesity.

It is possible that estrogens implicated in more frequent TLESRs (25) could skew the results of GERD-related symptoms in our predominantly female population (79% female versus 21% male in obese subjects and 78% female and 22% male in normal weight subjects) but age and gender corrected analysis of our data did not alter our results.

In keeping with published reports, our obese patients reported more frequent nausea, abdominal pain, bloating, diarrhea, and flatulence compared to normal weight individuals (9, 26). We did not distinguish between upper and lower abdominal pain as many patients had difficulty making this distinction. The mechanism of abdominal and bowel symptoms (Tables 1 and 3; Figure 1) is unclear and studies are few and data conflicting. It has been suggested that alteration in gastric emptying, gastric capacity, and gastric accommodation may play a role (27, 28). The role, if any of pro-inflammatory cytokines in abdominal pain, early satiety, flatulence, and diarrhea have not been studied. It is reasonable to assume that they do play a role in intestinal motility in general and not just in esophageal muscle contractility (23).

The prevalence of dysphagia has been reported to be approximately 10% and globus 8% in patients being evaluated for bariatric surgery (29). In our subjects, the prevalence of dysphagia to solids was 3% in obese subjects compared to 2% in normal weight individuals (*p* = 0.047). There is no obvious reason for the lower prevalence of dysphagia in our obese patients. Dysphagia to liquids, a symptom not reported in other studies, was higher in normal weight individuals although it did not reach statistical significance (*p* = 0.538).

Obese individuals in our study had similar prevalence of constipation and laxative use as normal weight individuals. The data in the literature are conflicting with regard to constipation where some studies have found no relationship between BMI and constipation (30), while others have found a higher prevalence of constipation in obese individuals (31) and those who were binge eaters (32).

Diarrhea, fecal urgency, and incontinence were more commonly reported in our obese subjects, a finding consistent with that reported in both population – as well as clinic-based studies (9, 29, 33). Decreased colonic compliance and colonic sensation in overweight individuals and decreased transit time in those with a BMI >30 kg/m^2^ could play a role in the pathogenesis of these symptoms (34).

The role of nutrient-sensing mechanisms in the GI tract (taste receptors) (35), their interplay with peptides such as glucagon-like peptide one, glucagon, neuropeptide Y, peptide YY, ghrelin, and oxyntomodulin (36) among others and their effect, if any, on satiety, gut motility, nutrient absorption, gut–brain signaling via vagal afferent neurons (37), and GI symptoms are yet to be explored.

There are no previous reports of oral symptoms in obese individuals compared to normal weight individuals. A sore tongue or a sensation of a swollen tongue (*p* < 0.015) and bleeding gums (*p* < 0.003) occurred more frequently in our obese patients. The increased sensation of a bitter taste or sour taste in the mouth is consistent with “water brash,” a symptom of gastroesophageal reflux, which occurs more frequently in obese individuals. The mechanism of the increased prevalence of oral symptoms could be related to micronutrient deficiencies, but this remains speculative since we did not measure any micronutrient level.

Our study has several strengths in that: (i) we were able to document the increased prevalence of GI symptoms in a clinic-based population, confirming the data on GI symptoms derived largely from population studies. (ii) We describe for the first time that oral symptoms (swollen tongue and bleeding gums) occur more frequently in obese patients. (iii) While there was a preponderance of females in our study, and females are known to have more functional bowel disease compared to males (38) even after adjusting for age and gender the results of our study do not change (Table 3). (iv) We did not cluster symptoms based on diagnosis so that clustering did not skew our data. For example, a higher irritable bowel syndrome score may be reflected in a higher abdominal pain score.

There are several limitations to our study: (i) we did not use validated questionnaires such as the gastroesophageal reflux questionnaire (8) or the bowel disease questionnaire (39). These questionnaires were designed to establish a diagnosis of gastroesophageal reflux or functional bowel disease in population cohorts; this was not the purpose of our study. Besides, our questionnaire did incorporate most of the questions found in the validated questionnaires. (ii) We did not evaluate anxiety and depression in our subjects. These conditions are associated with higher somatic complaints including GI symptoms. (iii) The prevalence of diabetes and vascular disease, among other chronic diseases, is higher in obesity. These diseases are known to be associated with abdominal symptoms and could have confounded our results. (iv) We did not take into account the influence of medications on GI symptoms used to treat the chronic diseases that these subjects had. (v) Our population was predominantly Caucasian, so the results of our study may not be generalizable to individuals of different ethnic backgrounds and possibly different food habits. (vi) A response rate of 28% in our study does not automatically imply that the frequencies are not accurate. They do have the potential to have more error but equally they could be accurate. Higher response rates of 54–68% are found in the literature in some studies. The higher response rate appears to be dependent on the level of education of the respondents. Other studies have shown a response rate of 26% for mail surveys, which is similar to the response rate in our study (40). This is in contrast to faxed and electronic surveys, which have higher response rates. It is important to bear in mind that the response rate indirectly reflects non-respondent bias. It has been suggested that more attention be paid to assessments of bias, and less to response rate (41). We therefore feel that our response rate does not detract from the validity of the study.

In conclusion, this study demonstrates that several GI symptoms are more commonly seen in obese individuals compared to normal weight individuals with similar comorbidities. Better understanding of the mechanism of these symptoms may be valuable in offering the appropriate treatment, i.e., surgical versus medical particularly as medical therapeutic options increase (36).

## Conflict of Interest Statement

The authors declare that the research was conducted in the absence of any commercial or financial relationships that could be construed as a potential conflict of interest.

## References

[1] Ng M Fleming T Robinson M Thomson B Graetz N Margono C Global, regional, and national prevalence of overweight and obesity in children and adults during 1980–2013: a systematic analysis for the Global Burden of Disease Study 2013. Lancet (2014) 384(9945):766–81. 10.1016/S0140-6736(14)60460-8 24880830 PMC4624264

[2] Olshansky SJ Passaro DJ Hershow RC Layden J Carnes BA Brody J A potential decline in life expectancy in the United States in the 21st century. N Engl J Med (2005) 352(11):1138–45. 10.1056/NEJMsr043743 15784668

[3] Centers for Disease Control and Prevention (CDC). National Center for Health Statistics. NCHS Obesity Data Available from: http://www.cdc.gov/nchs/data/factsheets/factsheet_obesity.htm

[4] Finkelstein EA Trogdon JG Cohen JW Dietz W. Annual medical spending attributable to obesity: payer-and service-specific estimates. Health Aff (Millwood) (2009) 28(5):w822–31. 10.1377/hlthaff.28.5.w822 19635784

[5] Flegal KM Graubard BI Williamson DF Gail MH. Excess deaths associated with underweight, overweight, and obesity. JAMA (2005) 293(15):1861–7. 10.1001/jama.293.15.1861 15840860

[6] Fontaine KR Redden DT Wang C Westfall AO Allison DB. Years of life lost due to obesity. JAMA (2003) 289(2):187–93. 10.1001/jama.289.2.187 12517229

[7] Wood GC Chu X Manney C Strodel W Petrick A Gabrielsen J An electronic health record-enabled obesity database. BMC Med Inform Decis Mak (2012) 12:45. 10.1186/1472-6947-12-45 22640398 PMC3508953

[8] Locke GR Talley NJ Weaver AL Zinsmeister AR. A new questionnaire for gastroesophageal reflux disease. Mayo Clin Proc (1994) 69(6):539–47. 10.1016/S0025-6196(12)62245-9 8189759

[9] Delgado-Aros S Locke GR III Camilleri M Talley NJ Fett S Zinsmeister AR Obesity is associated with increased risk of gastrointestinal symptoms: a population-based study. Am J Gastroenterol (2004) 99(9):1801–6. 10.1111/j.1572-0241.2004.30887.x 15330922

[10] Djarv T Wikman A Nordenstedt H Johar A Lagergren J Lagergren P. Physical activity, obesity and gastroesophageal reflux disease in the general population. World J Gastroenterol (2012) 18(28):3710–4. 10.3748/wjg.v18.i28.3710 22851863 PMC3406423

[11] Dutta SK Arora M Kireet A Bashandy H Gandsas A. Upper gastrointestinal symptoms and associated disorders in morbidly obese patients: a prospective study. Dig Dis Sci (2009) 54(6):1243–6. 10.1007/s10620-008-0485-6 18975090

[12] van Oijen MG Josemanders DF Laheij RJ van Rossum LG Tan AC Jansen JB. Gastrointestinal disorders and symptoms: does body mass index matter? Neth J Med (2006) 64(2):45–9. 16517988

[13] Anand G Katz PO Gastroesophageal reflux disease and obesity. Gastroenterol Clin North Am (2010) 39(1):39–46 10.1016/j.gtc.2009.12.002 20202577

[14] Locke GR III Talley NJ Fett SL Zinsmeister AR Melton LJ III. Risk factors associated with symptoms of gastroesophageal reflux. Am J Med (1999) 106(6):642–9. 10.1016/S0002-9343(99)00121-7 10378622

[15] Nocon M Labenz J Jaspersen D Meyer-Sabellek W Stolte M Lind T Association of body mass index with heartburn, regurgitation and esophagitis: results of the progression of gastroesophageal reflux disease study. J Gastroenterol Hepatol (2007) 22(11):1728–31. 10.1111/j.1440-1746.2006.04549.x 17914941

[16] Hampel H Abraham NS El-Serag HB. Meta-analysis: obesity and the risk for gastroesophageal reflux disease and its complications. Ann Intern Med (2005) 143(3):199–211. 10.7326/0003-4819-143-3-200508020-00006 16061918

[17] Corley DA Kubo A. Body mass index and gastroesophageal reflux disease: a systematic review and meta-analysis. Am J Gastroenterol (2006) 101(11):2619–28. 10.1111/j.1572-0241.2006.00849.x 16952280

[18] Lundell L Ruth M Sandberg N Bove-Nielsen M. Does massive obesity promote abnormal gastroesophageal reflux? Dig Dis Sci (1995) 40(8):1632–5. 10.1007/BF02212682 7648961

[19] O’Brien TF Jr Lower esophageal sphincter pressure (LESP) and esophageal function in obese humans. J Clin Gastroenterol (1980) 2(2):145–8 10.1097/00004836-198006000-00007 7440948

[20] Wu JC Mui LM Cheung CM Chan Y Sung JJ. Obesity is associated with increased transient lower esophageal sphincter relaxation. Gastroenterology (2007) 132(3):883–9. 10.1053/j.gastro.2006.12.032 17324403

[21] Sugerman HJ DeMaria EJ Felton WL III Nakatsuka M Sismanis A. Increased intra-abdominal pressure and cardiac filling pressures in obesity-associated pseudotumor cerebri. Neurology (1997) 49(2):507–11. 10.1212/WNL.49.2.507 9270586

[22] Pandolfino JE El-Serag HB Zhang Q Shah N Ghosh SK Kahrilas PJ. Obesity: a challenge to esophagogastric junction integrity. Gastroenterology (2006) 130(3):639–49. 10.1053/j.gastro.2005.12.016 16530504

[23] Rieder F Cheng L Harnett KM Chak A Cooper GS Isenberg G Gastroesophageal reflux disease-associated esophagitis induces endogenous cytokine production leading to motor abnormalities. Gastroenterology (2007) 132(1):154–65. 10.1053/j.gastro.2006.10.009 17241868

[24] Kato M Watabe K Hamasaki T Umeda M Furubayashi A Kinoshita K Association of low serum adiponectin levels with erosive esophagitis in men: an analysis of 2405 subjects undergoing physical check-ups. J Gastroenterol (2011) 46(12):1361–7. 10.1007/s00535-011-0453-3 21845377

[25] Nilsson M Johnsen R Ye W Hveem K Lagergren J. Obesity and estrogen as risk factors for gastroesophageal reflux symptoms. JAMA (2003) 290(1):66–72. 10.1001/jama.290.1.66 12837713

[26] Bernal-Reyes R Monzalvo Lopez A Bernal-Serrano M. [Prevalence of gastrointestinal symptoms in overweight and obese subjects: an epidemiologic study on a Mexican population]. Rev Gastroenterol Mex (2013) 78(1):28–34. 10.1016/j.rgmx.2012.10.006 23395528

[27] Hutson WR Wald A. Obesity and weight reduction do not influence gastric emptying and antral motility. Am J Gastroenterol (1993) 88(9):1405–9. 8362840

[28] Kim DY Camilleri M Murray JA Stephens DA Levine JA Burton DD. Is there a role for gastric accommodation and satiety in asymptomatic obese people? Obes Res (2001) 9(11):655–61. 10.1038/oby.2001.89 11707531

[29] Fysekidis M Bouchoucha M Bihan H Reach G Benamouzig R Catheline JM. Prevalence and co-occurrence of upper and lower functional gastrointestinal symptoms in patients eligible for bariatric surgery. Obes Surg (2012) 22(3):403–10. 10.1007/s11695-011-0396-z 21503810

[30] Pourhoseingholi MA Kaboli SA Pourhoseingholi A Moghimi-Dehkordi B Safaee A Mansoori BK Obesity and functional constipation; a community-based study in Iran. J Gastrointestin Liver Dis (2009) 18(2):151–5. 19565043

[31] Eslick GD. Prevalence and epidemiology of gastrointestinal symptoms among normal weight, overweight, obese and extremely obese individuals. Gastroenterol Clin North Am (2010) 39(1):9–22. 10.1016/j.gtc.2009.12.007 20202575

[32] Crowell MD Cheskin LJ Musial F. Prevalence of gastrointestinal symptoms in obese and normal weight binge eaters. Am J Gastroenterol (1994) 89(3):387–91. 8122651

[33] Talley NJ Quan C Jones MP Horowitz M. Association of upper and lower gastrointestinal tract symptoms with body mass index in an Australian cohort. Neurogastroenterol Motil (2004) 16(4):413–9. 10.1111/j.1365-2982.2004.00530.x 15305996

[34] Delgado-Aros S Camilleri M Garcia MA Burton D Busciglio I. High body mass alters colonic sensory-motor function and transit in humans. Am J Physiol Gastrointest Liver Physiol (2008) 295(2):G382–8. 10.1152/ajpgi.90286.2008 18617555 PMC2519862

[35] Depoortere I. Taste receptors of the gut: emerging roles in health and disease. Gut (2014) 63(1):179–90. 10.1136/gutjnl-2013-305112 24131638

[36] Troke RC Tan TM Bloom SR The future role of gut hormones in the treatment of obesity. Ther Adv Chronic Dis (2014) 5(1):4–14 10.1177/2040622313506730 24381724 PMC3871274

[37] Dockray GJ. Gastrointestinal hormones and the dialogue between gut and brain. J Physiol (Lond) (2014) 592(Pt 14):2927–41. 10.1113/jphysiol.2014.270850 24566540 PMC4214649

[38] Longstreth GF Thompson WG Chey WD Houghton LA Mearin F Spiller RC Functional bowel disorders. Gastroenterology (2006) 130(5):1480–91 10.1053/j.gastro.2005.11.061 16678561

[39] Talley NJ Phillips SF Wiltgen CM Zinsmeister AR Melton LJ III. Assessment of functional gastrointestinal disease: the bowel disease questionnaire. Mayo Clin Proc (1990) 65(11):1456–79. 10.1016/S0025-6196(12)62169-7 2232900

[40] Cobanoglu C Moreo P Warde B A comparison of mail, fax and web-based survey methods. Int J Market Res (2001) 43(4):441–52.

[41] Asch DA Jedrziewski MK Christakis NA. Response rates to mail surveys published in medical journals. J Clin Epidemiol (1997) 50(10):1129–36. 10.1016/S0895-4356(97)00126-1 9368521

